# Functional and Morphological Changes Induced in *Mytilus* Hemocytes by Selected Nanoparticles

**DOI:** 10.3390/nano11020470

**Published:** 2021-02-12

**Authors:** Manon Auguste, Craig Mayall, Francesco Barbero, Matej Hočevar, Stefano Alberti, Giacomo Grassi, Victor F. Puntes, Damjana Drobne, Laura Canesi

**Affiliations:** 1 Department of Environmental, Earth, and Life Sciences (DISTAV), University of Genoa, 16136 Genoa, Italy; Laura.Canesi@unige.it; 2 Biotechnical Faculty, University of Ljubljana, 1000 Ljubljana, Slovenia; craig_mayall@hotmail.co.uk (C.M.); Damjana.Drobne@bf.uni-lj.si (D.D.); 3 Institut Català de Nanociència i Nanotecnologia (ICN2), CSIC and The Barcelona Institute of Science and Technology (BIST), Campus UAB, Bellaterra, 08193 Barcelona, Spain; fra.barbero@gmail.com (F.B.); victor.puntes@icn2.cat (V.F.P.); 4 Institute of Metals and Technology (IMT), 1000 Ljubljana, Slovenia; matej.hocevar@imt.si; 5 Department of Chemistry and Industrial Chemistry, University of Genoa, 16136 Genoa, Italy; stefano.alberti@edu.unige.it; 6 Department of Physical, Earth, and Environmental Sciences, University of Siena, 53100 Siena, Italy; giacomograssi6@gmail.com

**Keywords:** hemocytes, *Mytilus*, in vitro, scanning electron microscopy, immune response

## Abstract

Nanoparticles (NPs) show various properties depending on their composition, size, and surface coating, which shape their interactions with biological systems. In particular, NPs have been shown to interact with immune cells, that represent a sensitive surveillance system of external and internal stimuli. In this light, in vitro models represent useful tools for investigating nano-bio-interactions in immune cells of different organisms, including invertebrates. In this work, the effects of selected types of NPs with different core composition, size and functionalization (custom-made PVP-AuNP and commercial nanopolystyrenes PS-NH_2_ and PS-COOH) were investigated in the hemocytes of the marine bivalve *Mytilus galloprovincialis*. The role of exposure medium was evaluated using either artificial seawater (ASW) or hemolymph serum (HS). Hemocyte morphology was investigated by scanning electron microscopy (SEM) and different functional parameters (lysosomal membrane stability, phagocytosis, and lysozyme release) were evaluated. The results show distinct morphological and functional changes induced in mussel hemocytes depending on the NP type and exposure medium. Mussel hemocytes may represent a powerful alternative in vitro model for a rapid pre-screening strategy for NPs, whose utilization will contribute to the understanding of the possible impact of environmental exposure to NPs in marine invertebrates.

## 1. Introduction

Due to the rapid expansion of production and use of nanoparticles (NPs) and their consequent release in different ecosystems, there is increasing concern on the utilization of alternative, affordable biological animal models for investigating nanosafety in environmental species. In vitro testing that focuses on specific cellular functions (i.e., immune responses) may provide an ideal starting point for developing a rapid prescreening strategy for NPs [1]. This would apply not only to vertebrate models, but also to invertebrates, which account for over 95% of animal species.

In the aquatic environment, which represents the final sink for all anthropogenic contaminants, including NPs, suspension-feeding invertebrates, due to their filtration ability for nutrition and respiration needs, have been identified as an unique target group for NP ecotoxicity [2,3,4,5]. In particular, in bivalve molluscs, abundant cell types involved in digestion or immune responses (digestive cells and hemocytes, respectively), have the capacity to internalize particles from the nano- to the micro-size via endocytic/phagocytic pathways [4,5,6].

The bivalve immune system relies solely on innate immunity, which mainly involves circulating hemocytes acting in collaboration with other soluble factors present in hemolymph serum [7,8]. This system responds very fast upon its encounter with foreign particles, making it a suitable tool for studying the effects of NPs using in vitro methods [9,10,11,12,13]. In particular, in the hemocytes of the marine mussel *Mytilus galloprovincialis*, the utilization of a battery of immune-related biomarkers has proven useful in the evaluation of the effects of a number of NPs, that can modulate responses with consequent immunotoxic effects or stimulation of immune parameters, leading to inflammation, depending on the NP type and on the conditions of exposure [12,14]. These studies underlined how mussel hemocytes represent a sensitive target for different NPs and their potential application as a starting point to more accurately design further studies that are relevant at the whole animal level.

The utilization of primary cells or cell lines is already established for vertebrates but these existing protocols need to be adapted and/or improved for bivalve in vitro assays [1,15,16]. Hemocytes freshly isolated from adult mussels (*M. galloprovincialis*) can be maintained in different media (artificial seawater, salt-enriched culture medium or hemolymph serum) for some hours, without impacting cell survival and biochemical/functional features [9,12,17,18]. All these media, characterized by a high ionic strength, will however affect NP behavior in terms of agglomeration and surface charge [19] as in the natural marine environment. Moreover, the behavior of NPs in mussel biological fluids (i.e., hemolymph serum) has been shown to be distinct for different types of NPs and important in determining interactions with hemocytes. In mussels, some types of NPs have been shown to associate with serum soluble components, organized into a “hard protein corona”, providing a specific biological identity for immune recognition and subsequent cellular responses [20,21]. All these factors are important to consider in the evaluation of the possible biological impact of NPs on the cells of marine invertebrates, to properly utilize these cellular in vitro models as a suitable alternative strategy for testing the immunosafety of NPs in environmental organisms.

In the present study, we investigated the in vitro responses of mussel hemocytes to different types of NPs in different exposure media with the aim of:

(a) investigating the sensitivity of hemocytes to NPs with different surface characteristics and which characteristic of NPs are most potent to provoke the hemocyte response in physiological medium;
(b) defining optimal experimental conditions for a sensitive, alternative, and affordable biological in vitro model for a rapid prescreening strategy for NPs.

Selected NPs were chosen on the basis of two criteria: first, the need to utilize a reference NP that is generally considered biologically inert as in mammalian model systems (i.e., AuNPs) [22,23]. To this aim, custom-made polyvinylpyrrolidone-PVP coated gold NPs (PVP-AuNPs), were designed to be stable in seawater medium and provide baseline information for further NPs testing. Second, the utilization of commercially available surface-modified NPs that could be utilized as model NPs of environmental interests (e.g., nanoplastics). Commercial surface modified polystyrene nanoplastics (amino- and carboxy-modified nanopolystyrene, PS-NH_2_ and PS-COOH) were utilized to provide an estimate of the role of different surface characteristics in hemocyte nano-bio-interactions.

The short-term in vitro effects of these selected NPs at different concentrations were compared in freshly isolated hemocytes from *M. galloprovincialis* exposed in either artificial seawater (ASW) or filtered hemolymph serum (HS). Upon exposure, the morphological changes induced in different experimental conditions were investigated by scanning electron microscopy (SEM). Complementary experiments were performed to measure different functional immune markers, from functional integrity of lysosomes (lysosomal membrane stability) and phagocytic ability to extracellular defense mechanisms (e.g., lysozyme release and the production of ROS-reactive oxygen species).

## 2. Materials and Methods

### 2.1. Nanoparticle Synthesis and Characterization

Custom-made PVP-AuNPs were synthesized by the Catalan Institute of Nanoscience and Nanotechnology (ICN2) (Barcelona, Spain), according to the previously reported seeded-growth methods. Detailed synthetic procedure can be found in Bastús et al. (2011) [24]. Tetrachloroauric (III) acid trihydrate (99.9% purity), sodium citrate tribasic dihydrate (≥99%), and polyvinylpyrrolidone (55 KDa) were purchased from Sigma-Aldrich (Madrid, Spain). Artificial seawater (ASW) was prepared at 35 ppt salinity, pH 7.9–8.1 [25,26]. In brief, AuNPs were synthesized using a sodium citrate aqueous solution, and different synthesis, postproduction, and characterization steps are reported in more detail in Figure S1. The AuNP seeds (~10 nm) were grown to obtain the desired size (~30 nm) and measured by scanning electron microscopy in transmission mode (STEM) (FEI Magellan 400 XHR, Hillsboro, OR, USA) (Figure S1A,B). AuNPs were further coated with PVP, followed by several washing steps. UV-vis analyses using a Carry 4000 spectrophotometer (Agilent, Santa Clara, CA, USA) were performed to verify the AuNP aggregation state and sample concentration (Figure S1C). The final hydrodynamic diameter (Figure S1D) and ζ-potential were determined by dynamic light scattering (DLS) and laser Doppler velocimetry using a Zetasizer Nano ZS instrument (Malvern Panalytical, Malvern, UK).

Commercial surface-modified nanopolystyrenes were from Bangs Laboratories, Inc. (Fishers, IN, USA). Amino-modified nanopolystyrene (PS-NH_2_), nominal size 50 nm, were previously characterized in different media (milliQ-MQ water, ASW, and HS) as described in Canesi et al. (2015, 2016) [11,12]. Carboxylated nanopolystyrene (PS-COOH), nominal size 60 nm, kindly provided by Ilaria Corsi’s lab (Univ. Siena, It), were from the same batch previously characterized in MQ water by DLS analysis [27]. In addition, in the present study, particle behavior was evaluated in suspensions of both ASW and *Mytilus* hemolymph serum (HS). The results on particle characterization are summarized in Table 1.

Possible contamination of NPs by lipopolysaccharides (LPS) was not of first concern, as mussels have shown to be resistant to LPS effects, due to their filtering ability and vicinity with human activities. In particular, mussel hemocytes in vitro show no sensitivity to LPS up to 10 µg/mL [28]. These concentrations are much higher than those of NP-associated LPS [29,30].

### 2.2. Mussels, Hemolymph Collection, Preparation of Hemolymph Serum and Hemocyte Monolayers and Exposure Conditions

Mussels (*Mytilus galloprovincialis* Lam.) were purchased from an aquaculture farm (Chioggia, IT), transferred to the laboratory, and acclimatized in static tanks containing aerated ASW (1 L/animal), 35 ppt, at 16 ± 1 °C for 24 h. For each sample, hemolymph was extracted from the adductor muscle of 4–5 animals, using a syringe via a noninvasive method, filtered with gauze, and pooled in Falcon tubes. To obtain hemolymph serum-HS (i.e., hemolymph free of cells), whole hemolymph was centrifuged at 900× *g* for 10 min, and the supernatant was passed through a 0.22 μm filter. All procedures were performed as previously described [11,12].

Hemocytes were incubated at 16 °C with different concentrations of NPs in ASW or HS, for 30 min to 1 h (depending on the functional parameter measured) as previously described [9,10,11,13]. Untreated (control in ASW or HS) hemocyte samples were run in parallel. The in vitro short-term exposure of mussel hemocytes has long been successfully applied to screen the effects of different types of NPs on immune function (see [9,10,11,13]). The underlying reason is that in these cells, induction of functional responses, as well as of stress responses and apoptotic processes, are particularly rapid, occurring within 1 h of exposure, in line with the physiological role of bivalve hemocytes as the first line of defense against non-self-material (see also [4]).

### 2.3. Hemocyte Morphology Using Scanning Electron Microscopy (SEM)

Hemocyte monolayers prepared on membrane filters (hydrophilic mixed cellulose esters membrane, Millipore©) of 3 μm pore size were incubated for 30 min with NP suspensions in ASW or HS. Control cells were run in parallel in ASW or HS medium. Cells were fixed for 2 h in 2.5% glutaraldehyde, 0.4% paraformaldehyde in modified PBS (100 mM phosphate-buffered saline adjusted to 450 mM osmolarity by addition of NaCl). Fixed cells were rinsed with the same buffer, postfixed in 1% osmium tetroxide in buffer for 1 h, and then stained with TOTO (thiocarbohydrazide/osmiumtetroxide/thiocarbohydrazide/osmiumtetroxide) conductive stain (adapted from [31,32]). Samples were then dehydrated by graded ethanol solutions and dried with hexamethyldisilizane (HMDS).

The dried samples were mounted on aluminum holders and sputter-coated with gold/palladium using precision etching coating system (Gatan 682, Pleasanton, CA, USA). Hemocytes were then examined with a field emission scanning electron microscope (SEM) (SEM, JEOL JSM-6500F, Tokyo, Japan) equipped with an energy dispersive X-ray spectroscopy (EDX) system. Samples exposed to AuNPs were sputter-coated with carbon in order to detect Au by EDX.

### 2.4. Hemocyte Functional Assays

Hemocyte functional parameters (lysosomal membrane stability, phagocytosis, lysosomal enzyme release, and extracellular ROS production) were evaluated essentially as previously described [9,11,13].

Lysosomal membrane stability (LMS) was evaluated by the NRR (neutral-red retention time) assay. Hemocyte monolayers (in triplicates) on glass slides were incubated with NP suspensions in filtered ASW or HS for 30 min and then incubated with a neutral-red (NR) solution (final concentration 40 µg/mL). Controls (unexposed samples) were run in parallel. The slides were examined under an optical microscope every 15 min until 50% of the cells showed sign of lysosomal leaking and the results are reported as percentage of control cells.

The percentage of phagocytic cell was evaluated by the uptake of neutral-red-stained zymosan on hemocyte monolayers. After 30 min of NP exposure, the neutral-red-stained zymosan in 0.05 M TrisHCl buffer (TBS) was added to the monolayers and left to incubate for 60 min. Monolayers were then washed three times with ASW, fixed with Baker’s formol calcium, and mounted in Kaiser’s glycerol gelatine medium for microscopical examination with an optical microscope. For each slide, the percentage of phagocytic hemocytes was calculated from a minimum of 200 cells in triplicates.

Extracellular ROS production was measured by the reduction of cytochrome c. The aliquots of hemolymph (in triplicates) were incubated with 500 μL of cytochrome c solution (75 mM ferricytochrome c in TBS buffer) in presence or not of NPs. The samples were read at 550 nm at different times (0, 30, and 60 min), and the results are expressed as changes in OD per mg of protein.

Lysozyme activity in the extracellular medium was measured in aliquots of serum. Whole hemolymph samples were incubated with or without NPs for 30 to 60 min. Lysozyme activity was determined spectrophotometrically at 450 nm utilizing *Micrococcus lysodeikticus* and was expressed as percentage of controls.

Total protein content was determined according to the Bradford method using bovine serum albumin (BSA) as a standard.

### 2.5. Statistics

Data are the mean ± SD of four independent experiments (n = 4), with each assay performed in triplicate. Data of functional parameters and hemocyte diameters were analyzed by one-way ANOVA followed by Tukey’s test at 95% confidence intervals (*p* ≤ 0.05). All statistical calculations were performed using the GraphPad Prism version 7.03 for Windows, GraphPad Software, San Diego, CA, USA.

## 3. Results

### 3.1. NP Characterization

Data on NP characteristics and behavior in exposure media are reported in Table 1.

Custom-made PVP-coated gold nanoparticles (PVP-AuNPs) in ASW were characterized by STEM to measure the size of the gold core and by DLS to obtain the hydrodynamic diameter. AuNPs had a gold core of 28.7 ± 3.2 nm (Figure S1B) and a PVP (55 KDa) coating leading to a final hydrodynamic diameter of 58.3 ± 0.16 nm (Figure S1D). PVP-AuNPs ζ-potential in ASW was −4.1 ± 0.6 mV.

For both nanopolystyrenes, in MQ the Z-average were in the same range as reported by the manufacturer, 57 ± 2 and 64.5 ± 0.6 nm for PS-NH_2_ and PS-COOH, respectively. The ζ-potential was positive (+42.8 ± 1 mV) for PS-NH_2_ and negative (−59.7 ± 2.6 mV) for PS-COOH [11,27]. In both ASW and HS, PS-NH_2_ showed a similar behavior, with formation of small agglomerates (~200 nm) and a lower ζ-potential (+14 mV) [11,12]. A distinct behavior was observed for PS-COOH, that in ASW formed large agglomerates (1822 ± 373.6 nm) and a less negative charge value (−26.9 mV). In HS, the size of the agglomerates was greatly reduced (189.1 ± 48.6 nm), and the ζ-potential showed even fewer negative values (−11.6 ± 1.5 mV).

For all NPs, PDI values were smaller in MQ that in ASW and HS, indicating a more homogenous dispersion in the absence of salts.

### 3.2. Effects of NPs on Hemocyte Morphology: SEM

The effects of different NPs on hemocyte morphology were investigated by SEM. For all NP types, the hemocytes were exposed for 30 min at a concentration of 10 μg/mL in ASW or HS suspension. Control hemocytes (in absence of NPs) were run in parallel in both media.

**Figure 1:**
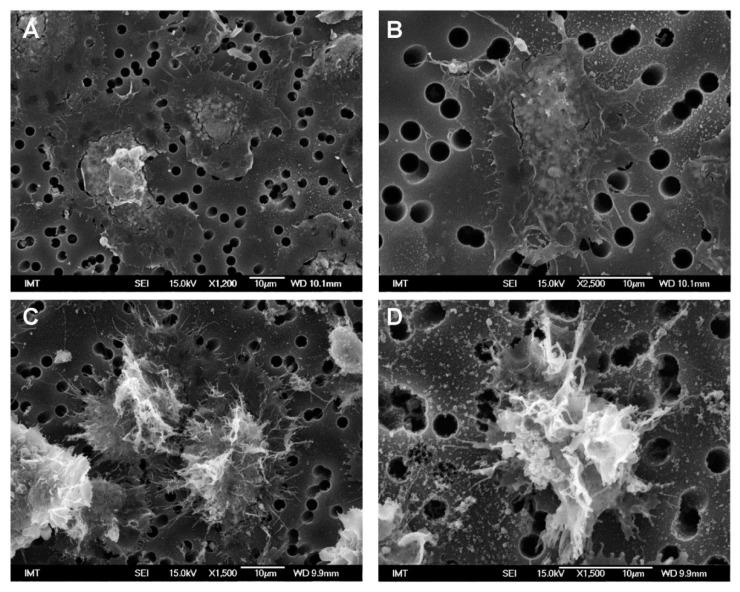
Scanning electron microscopy (SEM) images of control hemocytes from *M. galloprovincialis* in different media. (**A**,**B**) in artificial seawater (ASW); (**C**,**D**) in hemolymph serum (HS). Hemocytes were attached to a paper filter support with pore size of 3 µm.

Control hemocytes in ASW showed a characteristic extremely flat shape, with cells fully spread onto the filter support, with diameters ranging from 20 to 40 µm and showing a rather smooth surface, several short cell-surface extensions and absence of filopodia (Figure 1A,B). Due to the extreme flatness of the cells, fractures were often observed between the periphery and the center of the cells, which was thicker and contained granular structures presumably in the perinuclear region.

A different shape was observed in control hemocytes maintained in HS medium. They were not fully spread on the support and therefore thicker with smaller diameters (~20–28 µm). Their surface was irregular with several membrane ruffles and filopodia of variable shapes (Figure 1C,D).

**Figure 2:**
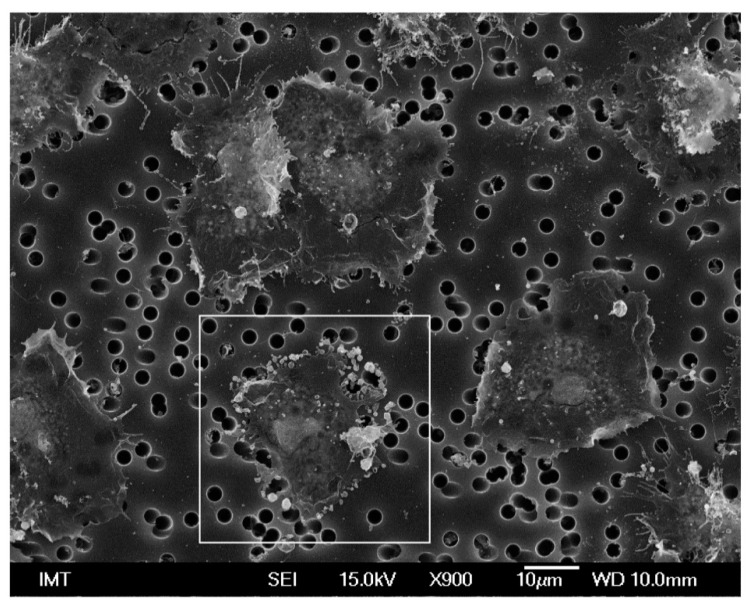
SEM images of hemocytes of *M. galloprovincialis* incubated for 30 min with PVP-AuNPs (10 µg/mL) in ASW suspension. In the framed cell, the presence of PVP-AuNPs was investigated using Energy-dispersive X-ray (EDX) analysis, and the details are presented in Figure S2.

After the addition of PVP-AuNP in ASW, cell morphology was similar to that of controls, even though some cells showed the presence of vesicles of micro-/submicro-metric size (≤1 µm diameter) on the edge of the plasma membrane (Figure 2). When the presence of Au was investigated by EDX, no gold was detected either inside the cells or in the surrounding environment (Figure S2). Similar observations were made in HS (not shown).

**Figure 3:**
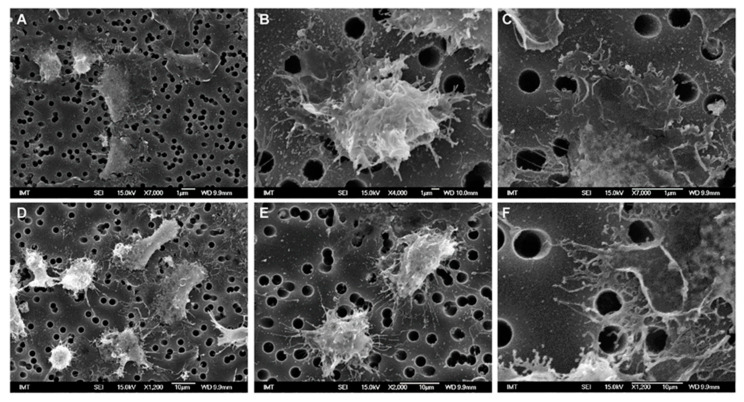
SEM images of hemocytes of *M. galloprovincialis* incubated for 30 min with PS-NH_2_ in ASW suspension (**A**,**B**) and in HS suspension (**D**,**E**). Details of membrane structures observed in ASW (**C**) and HS (**F**).

In contrast, PS-NH_2_ induced changes in hemocyte shape in both media (Figure 3). In ASW, some cells were still spread on the substrate, but they were not fully attached to the support, showing variable shapes (Figure 3A). Other hemocytes were smaller and thicker, showing the typical morphology of activated cells (Figure 3B). Such changes in shape were more evident in HS, where a large number of filopodia was observed (Figure 3D,E). Moreover, in both conditions, the edge of the cells often showed a complex lace-like pattern, due to a network of short filopodia (Figure 3C,F).

**Figure 4:**
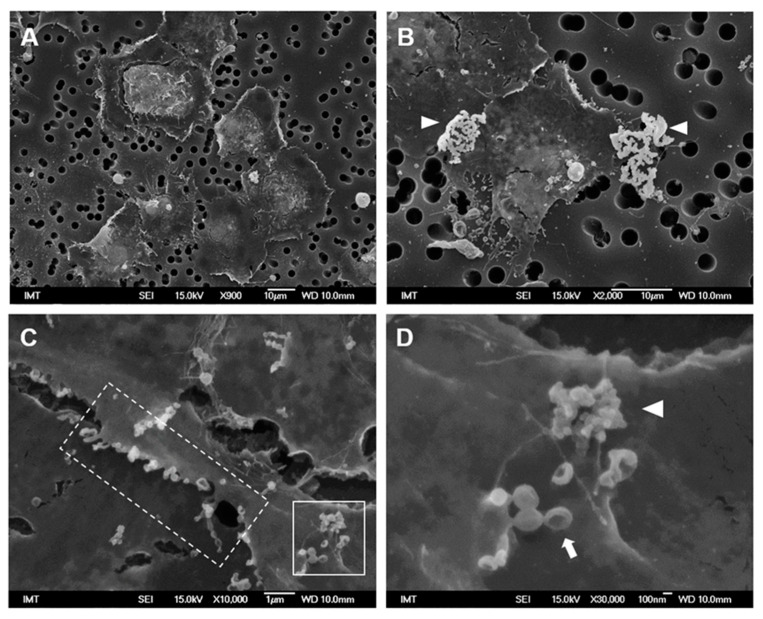
SEM images of *M. galloprovincialis* hemocytes incubated for 30 min with PS-COOH suspensions (10 µg/mL) in ASW (**A**). Arrowheads in (**B**) indicate large agglomerates of PS-COOH; (**C**) short extensions and vesicles along the plasma membrane (dotted frame); (**D**) Enlargement of the white frame in (**C**) indicating the presence of PS-COOH small agglomerates on the cell surface (arrowhead) as well as of concave vesicles (arrow).

Exposure to PS-COOH in ASW did not affect gross morphology of cells with respect to control hemocytes (Figure 4A). Large particle agglomerates were observed around some cells (arrowheads), appearing very white in contrast with the rest of the biological material (Figure 4B and Figure S3 for details of PS-COOH). Moreover, short membrane protrusions could be observed along the border of some cells, as well small extracellular vesicles (Figure 4C). The presence of small PS-COOH agglomerates on the cell surface was observed near vesicles with apparent concave shapes (Figure 4D).

**Figure 5:**
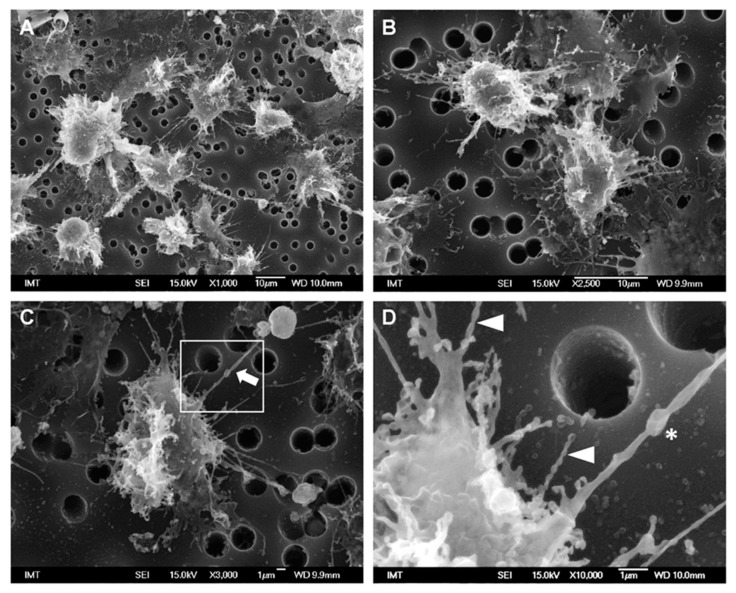
SEM images of *M. galloprovincialis* hemocytes incubated for 30 min with PS-COOH suspensions (10 µg/mL) in HS (**A**,**B**,**D**) Detail of cytoplasmic extensions observed in (**C**), indicating thicker filaments containing vesicles (asterisk), and filopodia with necklace-like structure (arrowheads).

In hemocytes exposed to PS-COOH in HS, cell morphology was apparently similar to those of control cells in HS; however, the formation of thin filopodia of about 3–8 μm in length was observed, apparently enabling communication between adjacent cells (Figure 5A,B). Among these, some longer (>15 μm) filopodia were observed (Figure 5C, arrow), sometimes apparently making contact with apoptotic cells/cell bodies. When observed in more detail (Figure 5D), these filaments appeared thicker (300–400 nm) and containing vesicles (asterisk). Moreover, other shorter filopodia appeared to be formed by a chain of vesicles connected by very thin necks, as in a necklace-like structure (arrowheads). In HS, no particle agglomerates were observed.

### 3.3. Hemocyte Functional Parameters

#### 3.3.1. PVP-AuNP

**Figure 6:**
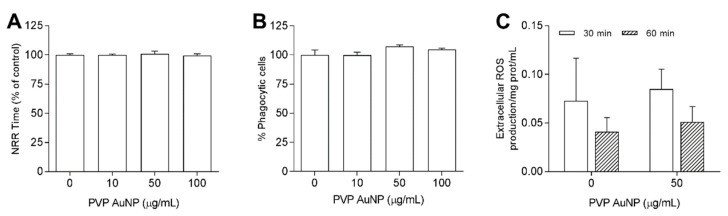
Effects of PVP-AuNPs in ASW on hemocyte: lysosomal membrane stability (LMS) (**A**), phagocytosis (**B**) and extracellular ROS production (**C**).

PVP-AuNP exposure in ASW did not affect hemocyte LMS and phagocytosis (Figure 6A,B) at any concentration tested up to 100 μg/mL (Figure 6A,B). Moreover, PVP-AuNPs did not affect extracellular ROS production up to 50 μg/mL (Figure 6C). Similarly, no effects were observed using HS as suspension medium (data not shown).

#### 3.3.2. Amino-Modified Nanopolystyrene—PS-NH_2_

**Figure 7:**
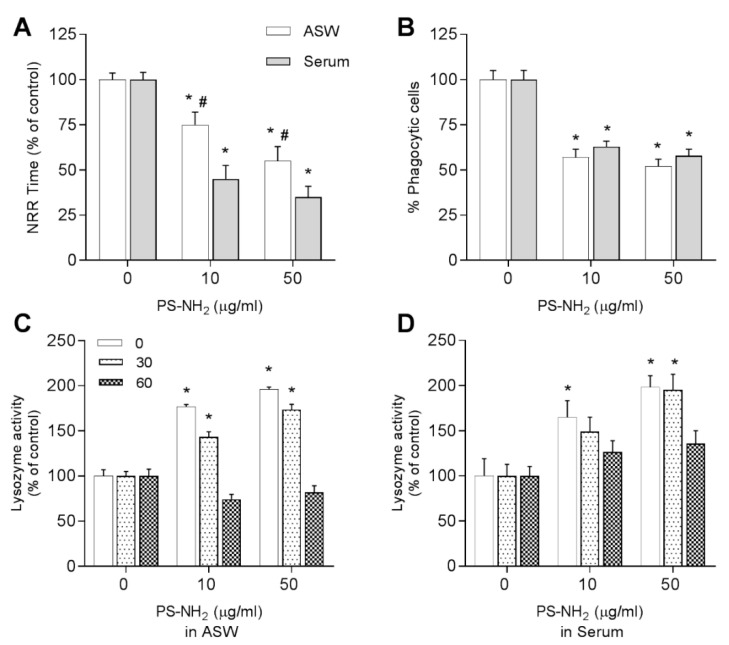
Effects of PS-NH_2_ in different suspension media in ASW (white bars) or hemolymph serum (HS) (grey bars) on mussel *M. galloprovincialis* hemocytes. (**A**) LMS; (**B**) phagocytosis; and lysozyme activity at time 0, after 30 and 60 min in ASW (**C**) and HS (**D**). Data, representing the mean ± SD of four experiments in triplicate, were analyzed by ANOVA followed by Tukey’s post hoc test, (*p* < 0.05): * = all treatments vs. controls; # = ASW vs. serum.

The effects of PS-NH_2_ on *Mytilus* hemocytes in different exposure media were previously reported in [11,12]. However, to mirror the effects observed for the changes in cell morphology, in this work further experiments were performed at the same concentration, 10 μg/mL in both media, and the results are reported in Figure 7.

A dose-dependent decrease in LMS was observed from 10 μg/mL in ASW, down to −50% at 50 μg/mL; a significantly stronger effect was observed in HS, at both concentrations (Figure 7A). The phagocytic activity showed a similar decrease (about −40% with respect to controls) at 10 and 50 µg/mL and in both media (Figure 7B). PS-NH_2_ also stimulated increase in lysozyme activity: the effect was transient in both media with highest values recorded directly immediately after NP addition and after 30 min exposure (Figure 7C,D).

#### 3.3.3. Carboxy-Modified Nanopolystyrene—PS-COOH

**Figure 8:**
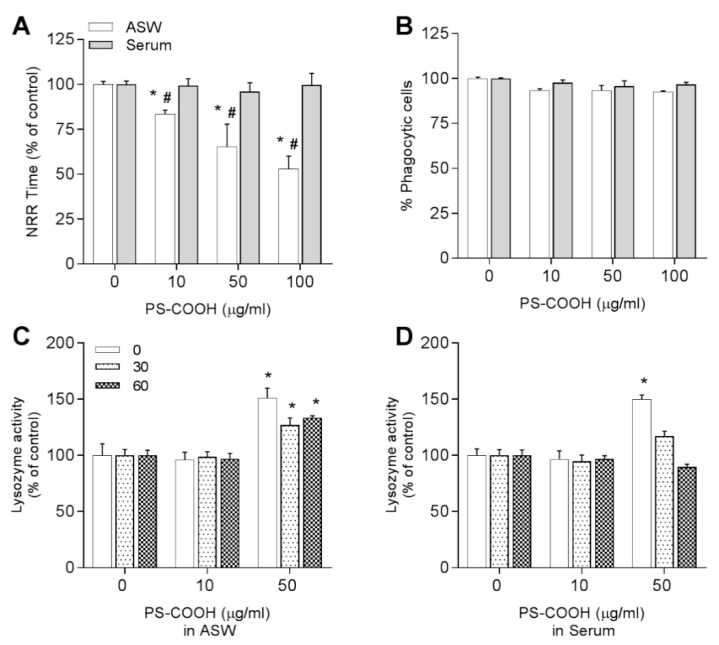
Effects of PS-COOH in different suspension media in ASW (white bars) or hemolymph serum-HS (grey bars) on mussel *M. galloprovincialis* hemocytes. LMS (**A**), phagocytosis (**B**), and lysozyme activity at time 0, after 30 and 60 min in ASW (**C**) and HS (**D**). Data representing the mean ± SD of four experiments in triplicate, were analyzed by ANOVA followed by Tukey’s post hoc test, (*p* < 0.05); * = all treatments vs. controls; # = ASW vs. serum.

The effects of PS-COOH are reported in Figure 8. In ASW suspensions, LMS was decreased from −10 to −45% with respect to controls, at concentrations ranging from 10 to 100 μg/mL, whereas no effects were observed in HS (Figure 8A). Phagocytic activity was unaffected in all experimental conditions (Figure 8B). Addition of PS-COOH immediately triggered lysozyme release by hemocytes in both media although only at 50 μg/mL. Interestingly, in ASW this rise in lysozyme activity was persistent up to 60 min exposure, whereas in HS a rapid decrease over time was observed (Figure 8C,D).

Because PS-COOH in HS showed distinct agglomeration and effects on hemocyte morphology, lysosomal membrane stability and time course of lysozyme release, its possible interactions with soluble serum components (i.e., the formation of a protein corona) were investigated. PS-COOH was incubated with hemocytes HS and isolation of the corona proteins was performed by centrifugation and 1D-gel electrophoresis as previously described for PS-NH_2_ and other NPs ([12,20] and Figure S4 for details). When samples were analyzed by gel electrophoresis (Figure S5), no detectable protein bands specific of the corona sample obtained with PS-COOH were observed, indicating the absence of proteins stably bound to the NPs (hard protein corona).

## 4. Discussion

In the present work, the in vitro interactions and effects of NPs in different media were investigated in the hemocytes of the marine mussel *M. galloprovincialis*. Custom-made PVP-AuNPs stable in ASW were first used as a reference material, and the results were compared with those obtained with two types of nanopolystyrene bearing distinct surface properties (PS-NH_2_ and PS-COOH). The results highlight distinct morphological and functional responses of mussel hemocytes depending on the core composition and chemicophysical properties of the NPs used and on exposure medium. To our knowledge, these are the first data on the effects of different NPs on detailed cell morphology evaluated by SEM coupled to evaluation of functional parameters in the immune cells of marine invertebrates.

Gold NPs represent a valuable tool for biomedical applications, i.e., diagnosis or targeted drug delivery, due to the peculiar properties of this material [33,34]. AuNPs appear generally inert toward biological systems, since they have been reported to poorly affect natural cell functions [22]. In particular, in mammalian systems, AuNPs have been shown to be relevant to meet the need for immunosafe particles for clinical purposes [23]. In this light, AuNPs can be utilized as a model to compare the interactions of NPs with innate immunity across different species. However, for a proper comparison, the main characteristics and behavior of NPs should be maintained in different experimental settings. Various NPs have been shown to strongly agglomerate in ASW, due to the high salt concentration which reduces the electrostatic repulsion of the particles [35]. To circumvent this problem, custom-made AuNPs, coated with PVP to maintain their dispersion in ASW, were successfully synthesized to allow for exposure of marine invertebrate cells in vitro.

Previous in vitro studies on *M. galloprovincialis* hemocytes exposed to citrate-coated AuNPs of different sizes (5, 15, 40 nm) for 24 h reported a slight decrease in cell viability at concentrations >50 μg/mL, with stronger effects caused by smaller-size AuNPs; however, the citrate coating used appeared to play a major role in the toxicity observed [16]. In gill explants of the clam *Ruditapes philippinarum*, the utilization of an optimized STEM-in-SEM methodology allowed for detection of AuNPs (24 nm) inside the gill cells, attached to the outer membrane of the mitochondria and to the nuclear envelope [36]. In the present work, no internalization of AuNPs was observed by *Mytilus* hemocytes; however, differences in phagocytic activity may be due to the cell type, the incubation time, or the AuNP characteristics. Moreover, the results here obtained show that short-term exposure to PVP-AuNPs did not affect the morphology or main functional parameters of mussel hemocytes. When the presence of Au was investigated by EDX, no gold was detected either inside the cells or in the surrounding environment. However, in some AuNP-exposed cells, small vesicles on the edge of the plasma membrane were observed, resembling ectosomes. Ectosomes are vesicles generated by the direct outward budding of the plasma membrane, which produces microvesicles, microparticles, and large vesicles in the size range of ~50 nm to 1 mm in diameter [37]. Human polymorphonuclear neutrophils (PMNs) upon activation release ectosomes which are characterized by the expression of phosphatidylserine and show anti-inflammatory/immunosuppressive activities toward macrophages [38]. In mussel hemocytes, the production of ectosomes and exchange of signaling molecules might represent an additional component of intercellular communication among different cell subpopulations, contributing to down-regulation of responses to AuNP exposure and immunoregulation as in human PMNs. However, this possibility requires further investigation.

Overall, these data indicate that AuNPs did not strongly interact with hemocytes and/or were lost during the fixing procedure. The data obtained in the present work confirm the suitability of the newly synthetized AuNP to be utilized as a negative control for testing the effects of NP exposure on *Mytilus* hemocytes, as well as any other marine invertebrate cell types. These data reinforce the concept of the importance of the choice of NPs and suitability of the protocols for handling marine invertebrate cells for in vitro testing of NP safety. Many parameters can influence the biological activity of NPs, including core composition, surface charge, and agglomeration state. To evaluate the effects of NP functionalization on *Mytilus* hemocytes, polystyrene NPs (PSNPs) of similar size (50–60 nm) but carrying different surface modifications (–NH_2_ and –COOH) were compared in vitro. PS-NH_2_ showed little agglomeration (~200 nm) in ASW, and slightly smaller (~178 nm) in HS medium, while retaining a similar positive surface charge (+14.2 mV) in both media [12]. PS-NH_2_ induced significant changes in functional parameters in both ASW and HS, with stronger effects observed in HS as previously described [11,12]. These results are supported by the present data here obtained by SEM on cell morphology in HS with respect to ASW.

The difference observed between the two media is in line with the reported formation of a stable biomolecular corona recorded for PS-NH_2_ in HS [12], whose unique protein component was identified as the extrapallial protein precursor (EPp) (also called MgC1q6). EPp is the most abundant serum protein encountered in *Mytilus* HS, and it is known to play a key role in the specific recognition of both selected bacterial strains and NP types [39,40]. The presence of a protein corona on PS-NH_2_ would likely increase the stability of NPs and contribute to reducing agglomeration, as well as to regulate nano-bio-interactions with hemocytes and the consequent outcome of the cellular response.

Distinct observations were made with PS-COOH, in terms of both particle behavior in exposure media and morphological and functional responses of hemocytes. In ASW, PS-COOH showed the formation of large agglomerates (>1500 nm) and retained a negative surface charge (−26 mV). Such a high agglomeration in ASW is likely to occur due to the interactions between the negative surface charge (-COO^−^) retained at pH ≥ 5 and high concentrations of divalent cations (e.g., Ca^2+^ and Mg^2+^) naturally present in seawater [41,42]. The results of SEM analysis indicate the absence of gross morphological changes in hemocytes exposed to PS-COOH at 10 µg/mL in ASW; accordingly, in these conditions, only a slight decrease in LMS was observed, whereas lysozyme release and phagocytosis were unaffected. This confirms that large NP agglomerates show little interactions with hemocytes.

When suspended in HS, PS-COOH formed smaller agglomerates (~180 nm) that were not detectable around the cells by SEM; in addition, under these experimental conditions, gross cell morphology and functional parameters were not significantly affected. However, SEM allowed for the detection of some subtle morphological changes, like formation of multiple filopodial extensions of variable length and shape that were not observed in all the other experimental conditions tested. The roles of filopodia can be various, from sensing or communication with the surrounding environment [43], to mobility, particle engulfment, and the production and release of molecules [44]. In mussel hemocytes exposed to PS-COOH in HS, we observed thicker filopodia containing vesicles, and/or shorter and thinner filopodia apparently formed by a chain of spherical vesicles connected by ultrathin necks. Some of them seemed to connect with the neighboring cell or with apoptotic bodies/vesicles. Drab et al. (2019) recently reported several mechanisms that could explain the presence and formation of these necklace-like structures, which can represent an intermediary stage in the formation of structures known as tunneling nanotubes (TNTs). TNTs are a new emerging cell-to-cell communication tool, that allows for the selective transfer of cellular components, signaling molecules, and pathogens between cells [45]. The presence and roles of TNTs have been mainly described in mammalian cells, including immune cells, where they play several roles in responding to stress, including the transport of nanomaterials [46,47]. Interestingly, we have recently reported the presence of similar structures exchanging lysosomal vesicles and mitochondria in live *M. galloprovincialis* hemocytes [48]. The results here obtained suggest that, under certain experimental conditions, NPs may induce TNT formation in invertebrate immune cells, thus probably allowing for an intercellular communication system that may participate in the protection against NP toxicity. Further work will be needed to confirm and define the presence of these peculiar structures in *Mytilus* hemocytes and appreciate their role in the effects that different NPs have on their immune function.

The results obtained with PS-COOH are the first data on the effects of negatively charged nanoplastics in the cells of marine bivalves. Data obtained in the presence of HS suggest that the observed effects may be due to the formation of a NP-protein corona, in analogy with previous data obtained with other NPs, including PS-NH_2_ and nano-oxides [20]. However, we could not identify any protein stably bound to PS-COOH (hard corona). These data do not exclude the possible interactions with other hemolymph proteins loosely bound to PS-COOH (soft corona) that may participate in modulating the responses of hemocytes and that cannot be detected with the method utilized. Grassi et al. (2019) studied the corona formation and composition in sea urchin coelomic fluid for PS-NH_2_ and PS-COOH and reported a similar hard-corona proteomic pattern for both PS-NP types [27]. The discrepancies with this work not only may be due to the profound differences in protein composition between biological fluids of sea urchins and mussels but also to the methodological approach using different protein separation (2D-electrophoresis). Finally, in mussel hemocytes, in contrast with data obtained with PS-NH_2_, where the presence of a stable protein corona elicited stronger effects/damages, the presence of proteins more loosely interacting with PS-COOH (soft corona) would participate in protective mechanisms, contributing to the biological response, as indicated by the results obtained measuring functional parameters.

Overall, the results further underline the importance of the surface charge, rather than the core material, in determining NP behaviour in exposure medium and consequent specificity of the nano-bio-interactions with mussel hemocytes leading to biological responses in vitro.

The application of SEM revealed for the first time the detailed morphology of hemocytes from *M. galloprovincialis* kept in different media and allowed for the evaluation of the morphological changes induced by different NPs in different experimental conditions. The results obtained on cell morphology are in line with those of determination of functional parameters, because the experimental conditions that elicited more evident morphological changes were always accompanied by the activation of stronger functional responses. The morphological and functional approach thus provides a better understanding of the results obtained when testing different types of NPs in vitro. This basal information will contribute to developing more standardized protocols for the in vitro screening of nanosafety in marine invertebrate cell models. This knowledge will also help better define protocols for in vivo exposure to NPs at environmentally realistic exposure concentrations.

In conclusion, the experimental system used in our study represents a suitable approach for fast evaluation of the biological activity of engineered nanomaterials in mussel immune cells. The most important characteristic of a prescreening tests is its capacity to discriminate among particles with different characteristics and what responses are biologically relevant. Our results confirm the specificity of the responses of mussel hemocytes (morphology and functional parameters) to different NPs.

## Figures and Tables

**Table 1:** Physicochemical characterization of NPs tested in vitro, PVP-AuNP, nanopolystyrenes (PS-NH_2_ and PS-COOH) behavior in exposure medium.

NPs	Medium	Z-Average (nm)	PDI	ζ-Potential (mV)
PVP-AuNP	ASW	58.3 ± 0.16	0.12 ± 0.01	−4.1 ± 0.6
PS-NH_2_	MQ ^1^	57 ± 2	0.07 ± 0.02	+42.8 ± 1
	ASW ^1^	200 ± 6	0.3 ± 0.02	+14.2 ± 2
	HS ^2^	178 ± 2	0.34 ± 0.05	+14.2 ± 1
PS-COOH	MQ ^3^	64.5 ± 0.6	0.06 ± 0.02	−59.7 ± 2.6
	ASW	1822 ± 373.6	0.228	−26.9 ± 1.9
	HS	189.1 ± 48.6	0.288	−11.6 ± 1.5

PDI = polydispersity index; ζ = zeta potential; MQ = MilliQ water; ASW= artificial seawater; HS = *Mytilus* hemolymph serum; ^1^ from [11]; ^2^ from [12]; ^3^ from [27].

## Supplementary Materials

The following are available online at https://www.mdpi.com/2079-4991/11/2/470/s1, Figure S1: Characterization of synthesis and postproduction steps of synthesized PVP-AuNPs; Figure S2: Energy-dispersive X-ray (EDX) analysis of a SEM sample of a hemocyte exposed to PVP-AuNPs in ASW; Figure S3: SEM images showing in more details PS-COOH suspensions in ASW; Figure S4: Schematic overview of the protocol utilized to identify the mussel PS-COOH corona (C) from *Mytilus galloprovincialis* hemolymph serum (HS); Figure S5: Separation of PS-COOH protein complexes from HS of *M. galloprovincialis* proteins by SDS-PAGE and staining with Coomassie Brilliant Blue.

## References

[1] Barrick A., Guillet C., Mouneyrac C., Châtel A. (2018). Investigating the Establishment of Primary Cultures of Hemocytes from Mytilus Edulis. Cytotechnology.

[2] Canesi L., Ciacci C., Fabbri R., Marcomini A., Pojana G., Gallo G. (2012). Bivalve Molluscs as a Unique Target Group for Nanoparticle Toxicity. Mar. Environ. Res..

[3] Rocha T.L., Gomes T., Sousa V.S., Mestre N.C., Bebianno M.J. (2015). Ecotoxicological Impact of Engineered Nanomaterials in Bivalve Molluscs: An Overview. Mar. Environ. Res..

[4] Canesi L., Corsi I. (2016). Effects of Nanomaterials on Marine Invertebrates. Sci. Total Environ..

[5] Canesi L., Auguste M., Bebianno M.J. (2019). Sublethal effects of nanoparticles on aquatic invertebrates, from molecular to organism level. Ecotoxicology of Nanoparticles in Aquatic Systems.

[6] Moore M.N. (2006). Do Nanoparticles Present Ecotoxicological Risks for the Health of the Aquatic Environment?. Environ. Int..

[7] Allam B., Raftos D. (2015). Immune Responses to Infectious Diseases in Bivalves. J. Invertebr. Pathol..

[8] Gerdol M., Gomez-Chiari M., Castillo M.G., Figueras A., Fiorito G., Moreira R., Novoa B., Pallavicini A., Ponte G., Roumbedakis K. (2018). Immunity in molluscs: Recognition and effector mechanisms, with a focus on bivalvia. Advances in Comparative Immunology.

[9] Ciacci C., Canonico B., Bilaniĉovă D., Fabbri R., Cortese K., Gallo G., Marcomini A., Pojana G., Canesi L. (2012). Immunomodulation by Different Types of N-Oxides in the Hemocytes of the Marine Bivalve Mytilus Galloprovincialis. PLoS ONE.

[10] Canesi L., Ciacci C., Vallotto D., Gallo G., Marcomini A., Pojana G. (2010). In Vitro Effects of Suspensions of Selected Nanoparticles (C60 Fullerene, TiO_2_, SiO_2_) on Mytilus Hemocytes. Aquat. Toxicol..

[11] Canesi L., Ciacci C., Bergami E., Monopoli M.P., Dawson K.A., Papa S., Canonico B., Corsi I. (2015). Evidence for Immunomodulation and Apoptotic Processes Induced by Cationic Polystyrene Nanoparticles in the Hemocytes of the Marine Bivalve Mytilus. Mar. Environ. Res..

[12] Canesi L., Ciacci C., Fabbri R., Balbi T., Salis A., Damonte G., Cortese K., Caratto V., Monopoli M.P., Dawson K. (2016). Interactions of Cationic Polystyrene Nanoparticles with Marine Bivalve Hemocytes in a Physiological Environment: Role of Soluble Hemolymph Proteins. Environ. Res..

[13] Auguste M., Ciacci C., Balbi T., Brunelli A., Caratto V., Marcomini A., Cuppini R., Canesi L. (2018). Effects of Nanosilver on Mytilus Galloprovincialis Hemocytes and Early Embryo Development. Aquat. Toxicol..

[14] Katsumiti A., Gilliland D., Arostegui I., Cajaraville M.P. (2015). Mechanisms of Toxicity of Ag Nanoparticles in Comparison to Bulk and Ionic Ag on Mussel Hemocytes and Gill Cells. PLoS ONE.

[15] Canesi L., Ciacci C., Balbi T. (2016). Invertebrate Models for Investigating the Impact of Nanomaterials on Innate Immunity: The Example of the Marine Mussel Mytilus Spp.. Curr. Bionanotechnol..

[16] Katsumiti A., Arostegui I., Oron M., Gilliland D., Valsami-Jones E., Cajaraville M.P. (2015). Cytotoxicity of Au, ZnO and SiO_2_ NPs Using In Vitro Assays with Mussel Hemocytes and Gill Cells: Relevance of Size, Shape and Additives. Nanotoxicology.

[17] Canesi L., Frenzilli G., Balbi T., Bernardeschi M., Ciacci C., Corsolini S., Della Torre C., Fabbri R., Faleri C., Focardi S. (2014). Interactive Effects of N-TiO2 and 2,3,7,8-TCDD on the Marine Bivalve Mytilus Galloprovincialis. Aquat. Toxicol..

[18] Katsumiti A., Thorley A.J., Arostegui I., Reip P., Valsami-Jones E., Tetley T.D., Cajaraville M.P. (2018). Cytotoxicity and Cellular Mechanisms of Toxicity of CuO NPs in Mussel Cells in Vitro and Comparative Sensitivity with Human Cells. Toxicol. Vitr..

[19] Bruinink A., Wang J., Wick P. (2015). Effect of Particle Agglomeration in Nanotoxicology. Arch. Toxicol.

[20] Canesi L., Balbi T., Fabbri R., Salis A., Damonte G., Volland M., Blasco J. (2017). Biomolecular Coronas in Invertebrate Species: Implications in the Environmental Impact of Nanoparticles. NanoImpact.

[21] Barbero F., Russo L., Vitali M., Piella J., Salvo I., Borrajo M.L., Busquets-Fité M., Grandori R., Bastús N.G., Casals E. (2017). Formation of the Protein Corona: The Interface between Nanoparticles and the Immune System. Semin. Immunol..

[22] Connor E.E., Mwamuka J., Gole A., Murphy C.J., Wyatt M.D. (2005). Gold Nanoparticles Are Taken Up by Human Cells but Do Not Cause Acute Cytotoxicity. Small.

[23] Sperling R.A., Rivera Gil P., Zhang F., Zanella M., Parak W.J. (2008). Biological Applications of Gold Nanoparticles. Chem. Soc. Rev..

[24] Bastús N.G., Comenge J., Puntes V. (2011). Kinetically Controlled Seeded Growth Synthesis of Citrate-Stabilized Gold Nanoparticles of up to 200 Nm: Size Focusing versus Ostwald Ripening. Langmuir.

[25] La Roche G., Eisler R., Tarzwell C. (1970). Bioassay Procedure for Oil and Oil Dispersant Toxicity Evaluation. J. Water Pollut Control. Fed.

[26] (2013). ASTM D1141-98 Standard Practice for the Preparation of Substitute Ocean Water.

[27] Grassi G., Landi C., Della Torre C., Bergami E., Bini L., Corsi I. (2019). Proteomic Profile of the Hard Corona of Charged Polystyrene Nanoparticles Exposed to Sea Urchin *Paracentrotus Lividus* Coelomic Fluid Highlights Potential Drivers of Toxicity. Environ. Sci. Nano.

[28] Hernroth B. (2003). The Influence of Temperature and Dose on Antibacterial Peptide Response against Lipopolysaccharide in the Blue Mussel, Mytilus Edulis. Fish. Shellfish Immunol..

[29] Li Y., Boraschi D. (2016). Endotoxin Contamination: A Key Element in the Interpretation of Nanosafety Studies. Nanomedicine.

[30] Li Y., Shi Z., Radauer-Preiml I., Andosch A., Casals E., Luetz-Meindl U., Cobaleda M., Lin Z., Jaberi-Douraki M., Italiani P. (2017). Bacterial Endotoxin (Lipopolysaccharide) Binds to the Surface of Gold Nanoparticles, Interferes with Biocorona Formation and Induces Human Monocyte Inflammatory Activation. Nanotoxicology.

[31] Drobne D., Sousa A.A., Kruhlak M.J. (2013). 3D Imaging of Cells and Tissues by Focused Ion Beam/Scanning Electron Microscopy (FIB/SEM). Nanoimaging.

[32] Millaku A., Drobne D., Torkar M., Novak S., Remškar M., Pipan-Tkalec Ž. (2013). Use of Scanning Electron Microscopy to Monitor Nanofibre/Cell Interaction in Digestive Epithelial Cells. J. Hazard. Mater..

[33] Tiwari P., Vig K., Dennis V., Singh S. (2011). Functionalized Gold Nanoparticles and Their Biomedical Applications. Nanomaterials.

[34] Yeh Y.-C., Creran B., Rotello V.M. (2012). Gold Nanoparticles: Preparation, Properties, and Applications in Bionanotechnology. Nanoscale.

[35] Bundschuh M., Filser J., Lüderwald S., McKee M.S., Metreveli G., Schaumann G.E., Schulz R., Wagner S. (2018). Nanoparticles in the Environment: Where Do We Come from, Where Do We Go To?. Environ. Sci. Eur..

[36] García-Negrete C.A., Jiménez de Haro M.C., Blasco J., Soto M., Fernández A. (2015). STEM-in-SEM High Resolution Imaging of Gold Nanoparticles and Bivalve Tissues in Bioaccumulation Experiments. Analyst.

[37] Kalluri R., LeBleu V.S. (2020). The Biology, Function, and Biomedical Applications of Exosomes. Science.

[38] Eken C., Sadallah S., Martin P.J., Treves S., Schifferli J.A. (2013). Ectosomes of Polymorphonuclear Neutrophils Activate Multiple Signaling Pathways in Macrophages. Immunobiology.

[39] Oliveri C., Peric L., Sforzini S., Banni M., Viarengo A., Cavaletto M., Marsano F. (2014). Biochemical and Proteomic Characterisation of Haemolymph Serum Reveals the Origin of the Alkali-Labile Phosphate (ALP) in Mussel (Mytilus Galloprovincialis). Comp. Biochem. Physiol. Part. D: Genom. Proteom..

[40] Pezzati E., Canesi L., Damonte G., Salis A., Marsano F., Grande C., Vezzulli L., Pruzzo C. (2015). Susceptibility of *V Ibrio Aestuarianu* s 01/032 to the Antibacterial Activity of *M Ytilus* Haemolymph: Identification of a Serum Opsonin Involved in Mannose-Sensitive Interactions: *Vibrio Aestuarianus* and Bivalve Haemocytes. Environ. Microbiol.

[41] Tallec K., Blard O., González-Fernández C., Brotons G., Berchel M., Soudant P., Huvet A., Paul-Pont I. (2019). Surface Functionalization Determines Behavior of Nanoplastic Solutions in Model Aquatic Environments. Chemosphere.

[42] Zhang F., Wang Z., Wang S., Fang H., Wang D. (2019). Aquatic Behavior and Toxicity of Polystyrene Nanoplastic Particles with Different Functional Groups: Complex Roles of PH, Dissolved Organic Carbon and Divalent Cations. Chemosphere.

[43] Heckman C.A., Plummer H.K. (2013). Filopodia as Sensors. Cell. Signal..

[44] Gauthier N.C., Masters T.A., Sheetz M.P. (2012). Mechanical Feedback between Membrane Tension and Dynamics. Trends Cell Biol..

[45] Drab M., Stopar D., Kralj-Iglič V., Iglič A. (2019). Inception Mechanisms of Tunneling Nanotubes. Cells.

[46] Sisakhtnezhad S., Khosravi L. (2015). Emerging Physiological and Pathological Implications of Tunneling Nanotubes Formation between Cells. Eur. J. Cell Biol..

[47] Korenkova O., Pepe A., Zurzolo C. (2020). Fine Intercellular Connections in Development: TNTs, Cytonemes, or Intercellular Bridges?. CST.

[48] Auguste M., Balbi T., Ciacci C., Canesi L. (2020). Conservation of Cell Communication Systems in Invertebrate Host–Defence Mechanisms: Possible Role in Immunity and Disease. Biology.

