# Supplementary material for: Functional and Morphological Changes Induced in Mytilus Hemocytes by Selected Nanoparticles
Source: Nanomaterials (Basel). 2021 Feb 12;11(2):470. doi: 10.3390/nano11020470 (PMC7918069; doi:10.3390/nano11020470)
Supplement: Supplementary file 1 [file nanomaterials-11-00470-s001.pdf]

nanomaterials

Article

Functional and Morphological Changes Induced in *Mytilus* Hemocytes by Selected Nanoparticles

Manon Auguste [1,*], Craig Mayall [2], Francesco Barbero [3], Matej Hočevar [4], Stefano Alberti [5], Giacomo Grassi [6], Victor F. Puntes [3], Damjana Drobne [2], and Laura Canesi [1]

1 Department of Environmental, Earth, and Life Sciences (DISTAV), University of Genoa, Genoa, Italy; Laura.Canesi@unige.it
2 Biotechnical Faculty, University of Ljubljana, Ljubljana, Slovenia; craig_mayall@hotmail.co.uk (C.M.); Damjana.Drobne@bf.uni-lj.si (D.D.)
3 Institut Català de Nanociència i Nanotecnologia (ICN2), CSIC and The Barcelona Institute of Science and Technology (BIST), Campus UAB, Bellaterra, Barcelona, Spain; fra.barbero@gmail.com (F.B.); victor.puntes@icn2.cat (V.P.)
4 Institute of Metals and Technology (IMT), Ljubljana, Slovenia; matej.hocevar@imt.si
5 Department of Chemistry and Industrial Chemistry, University of Genoa, Genoa, Italy; stefano.alberti@edu.unige.it
6 Department of Physical, Earth, and Environmental Sciences, University of Siena, Siena, Italy; giacomograssi6@gmail.com
* Correspondence: manon.auguste@edu.unige.it

Methods

Synthesis of PVP-AuNPs

A 150 ml of sodium citrate aqueous solution (2.2 mM) was brought to a boil in a three-neck flask under reflux, and subsequently injected with 1 ml of 25 mM of chloroauric acid $HAuCl_4$. After few minutes the solution became reddish, indicating AuNP formation (~ 10 nm, seeds); afterwards, different sequential steps of growth, consisting of sample dilution plus further addition of gold precursor led to the desired AuNPs size (~30 nm).

For the PVP NP coating procedure, 5 ml of citrate-stabilized AuNP (0.170 mg/ml) were dispersed drop wise in a stirred 45 ml solution of PVP (2 mM) in ASW. The solution was kept stirring overnight. PVP-AuNPs were purified from excess PVP and citrate by simple centrifugation (15.000 x g), supernatant removal, and further resuspension. During each washing step, 90% of the supernatant was removed, and the pellets were reconstituted to the initial volume with ASW. The procedure was repeated five times. After the last centrifugation step, 98% of the supernatant was removed and the pellet was not resuspended in order to keep the sample concentrated. UV-vis analysis showed no signs of NP aggregation after all the coating and purification/concentration procedures. The result of the UV-vis quantification analysis showed that the final PVP-AuNP sample was about 0.8 mg/mL. For detailed method see [1].

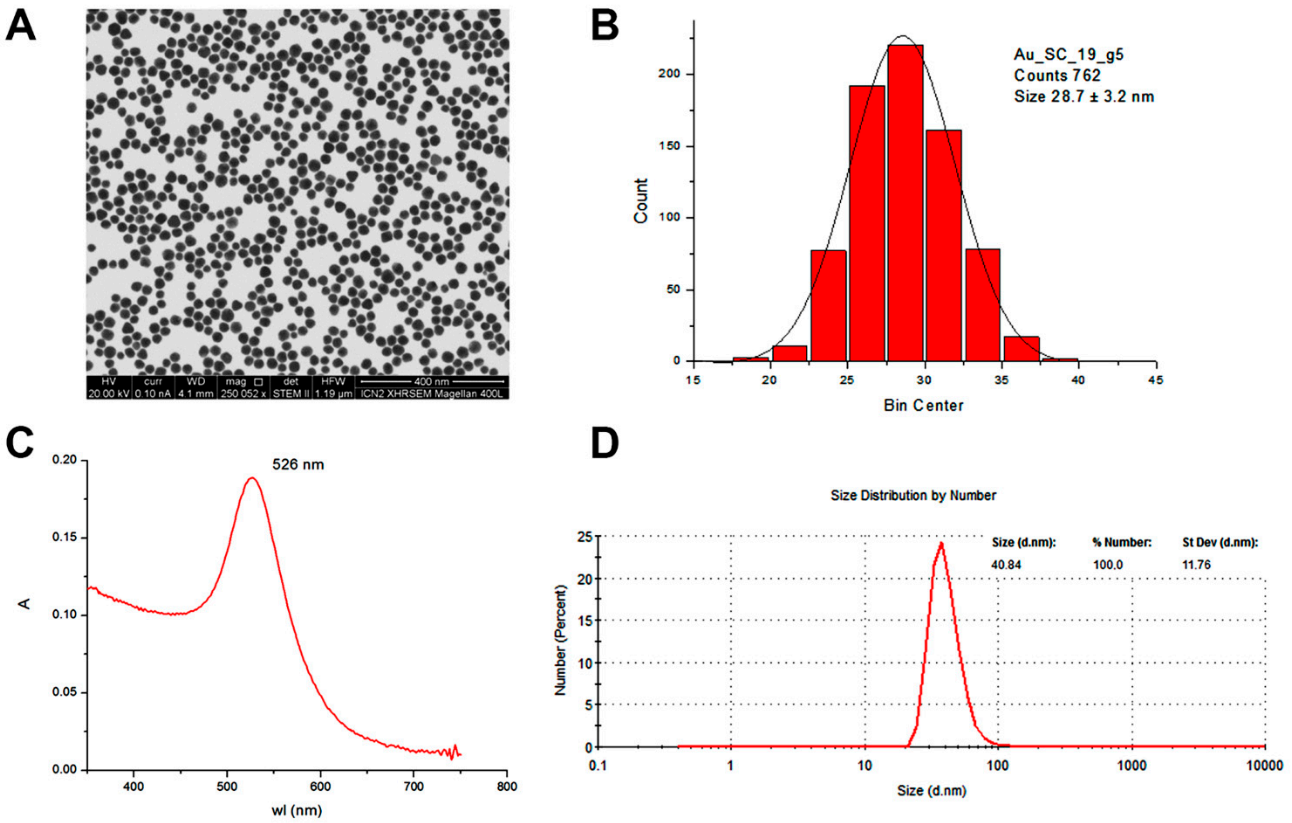

Figure S1. Characterization of synthesis and post-production steps of synthesized PVP-AuNPs.

Characterization of AuNPs core: A) STEM micrographs; B) Size distribution of AuNPs. Particle structural characterization was carried out using a Scanning Electron Microscopy (SEM) with FEI Magellan XHR SEM, in transmission mode (STEM) operated at 20 kV. A total of 762 particles from different regions of the grid were counted. Samples were prepared by drop-casting 3 µL of the sample on a carbon-coated copper TEM grid and left to dry under mild vacuum conditions.

Characterization of PVP-AuNP suspension in ASW: C) UV-visible spectra of PVP-AuNPs suspension in ASW. Analysis was carried out with a Shimadzu UV-2400 spectrophotometer at room temperature. D) Particle hydrodynamic diameter and Z-potential were determined by Dynamic Light Scattering, and Laser Doppler Velocimetry respectively, using a Malvern Zetasizer Nano ZS instrument equipped with a light source wavelength of 638.2 nm and a fixed scattering angle of 173°. Diameters are reported as Z-average and poly dispersity index (PDI) calculated by cumulant method analysis.

Results

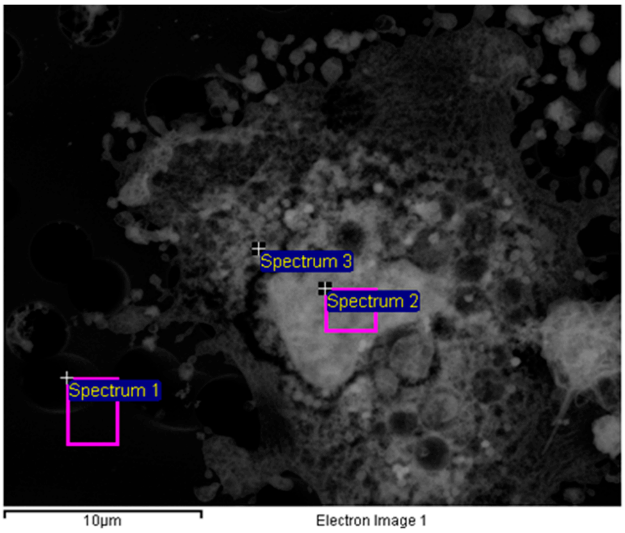

**Spectrum**	In stats.	C	O	Si	S	Os	**Au**	Total
**Spectrum 1**	Yes	76.77	15.73	0.00	0.31	7.18	0.00	100.00
**Spectrum 2**	Yes	29.86	16.52	1.42	1.07	51.13	0.00	100.00
**Spectrum 3**	Yes	38.33	17.79	0.86	1.09	41.92	0.00	100.00
Mean		48.32	16.68	0.76	0.83	33.41	0.00	100.00
Std. deviation		25.00	1.04	0.71	0.44	23.18	0.00	
Max.		76.77	17.79	1.42	1.09	51.13	0.00	
Min.		29.86	15.73	0.00	0.31	7.18	0.00	

Figure S2. Energy-dispersive X-ray (EDX) analysis of a SEM sample of a hemocyte exposed to PVP-AuNPs in ASW (10µg/ml; 30min) from Figure. 2. Frames indicate selected areas analyzed and the table the corresponding values of the elemental composition for each individual point. The results indicate the absence of Au in all the different areas analyzed.

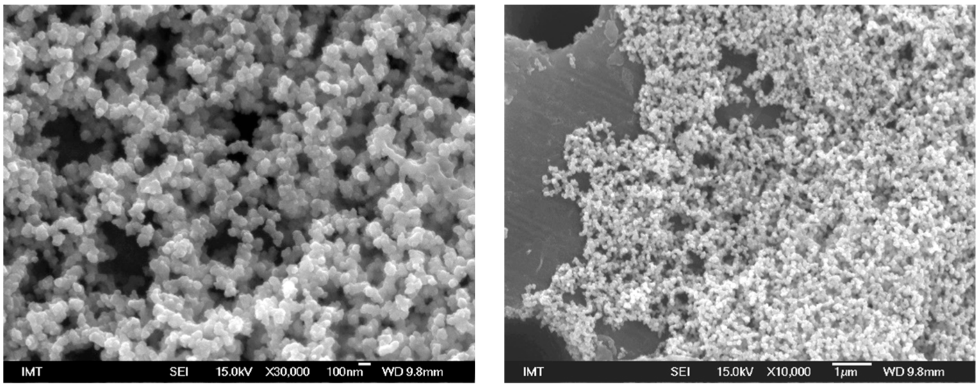

Figure S3. SEM images showing in more details PS-COOH suspensions in ASW.

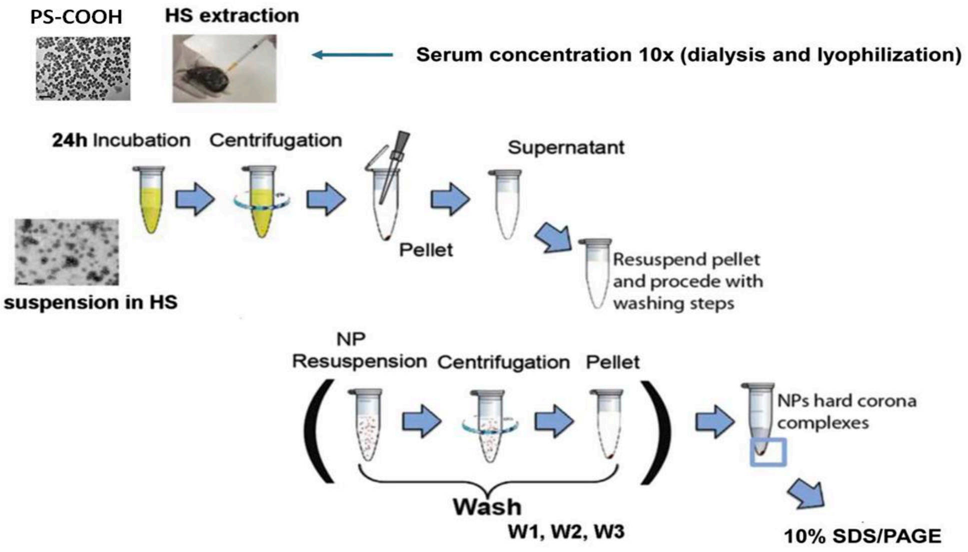

Figure S4. Schematic overview of the protocol utilized to identify the mussel PS-COOH corona (C) from *Mytilus galloprovincialis* hemolymph serum –HS (modified from [2]).

After hemolymph drawing from mussel adductor sinus, HS was obtained by filtration on 0.22 mm pore filters, dialyzed overnight against milliQ water to remove excess NaCl, and lyophilized. PS-COOH were incubated with 10x concentrated HS at the nominal concentration of 25 μg NP/mg protein/mL for 24 h at 18 °C under gentle shaking. After incubation, particle protein complexes were recovered by centrifugal isolation (adapted from [3]). Briefly, samples were centrifuged at 17,000 ×g for 75 min at 4 °C. The supernatant was stored at −80 °C (SN) and the pellet was re-suspended in ASW, transferred to a new vial, and centrifuged again at 17,000 ×g for 75 min to pellet the particle-protein complexes. This washing procedure used for removing unbound and loosely bound proteins from NPs, was repeated three times, to obtain W1, W2 and W3 samples. The pellet, containing the corona (C) proteins, was re-suspended in 0.1 mL ASW and protein content was evaluated, as well as in SN, W1, W2, W3 samples by the Bradford method. W3 samples did not contain any detectable amount of proteins. Samples were added with SDS-sample buffer and boiled for 5 min. Proteins (10 μg) were separated by 10% SDS/PAGE for direct visualization and comparison of stained protein patterns (see Fig. S5).

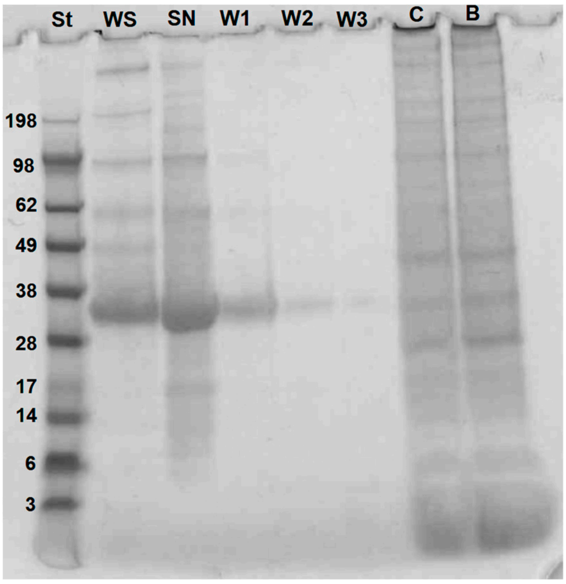

Figure S5. Separation of PS-COOH protein complexes from HS of *M. galloprovincialis* proteins by SDS-PAGE and staining with Coomassie Brilliant Blue. Lane 1: St = molecular weight standards. Lane 2: WS = whole serum sample in the absence of NPs (before all the purification and washing steps). Lane 3: SN = supernatant after the first centrifugation of the PS-COOH suspension in HS. Lanes 4–6: W1, W2, W3 = samples corresponding to the three washing steps. Lane 7: C = long-lived, hard corona proteins. Lane 8: B = blank sample of HS run in parallel in the absence of NPs that passed all corona isolation procedure. Note the absence of any difference in protein composition between sample C and B.

References

1. Bastús, N.G.; Comenge, J.; Puntes, V. Kinetically Controlled Seeded Growth Synthesis of Citrate-Stabilized Gold Nanoparticles of up to 200 nm: Size Focusing versus Ostwald Ripening. *Langmuir* **2011**, *27*, 11098–11105, doi:10.1021/la201938u.
2. Canesi, L.; Balbi, T.; Fabbri, R.; Salis, A.; Damonte, G.; Volland, M.; Blasco, J. Biomolecular coronas in invertebrate species: Implications in the environmental impact of nanoparticles. *NanoImpact* **2017**, *8*, 89–98, doi:10.1016/j.impact.2017.08.001.
3. Monopoli, M.P.; Pitek, A.S.; Lynch, I.; Dawson, K.A. Formation and Characterization of the Nanoparticle–Protein Corona. In *Nanomaterial Interfaces in Biology*; Bergese, P., Hamad-Schifferli, K., Eds.; Methods in Molecular Biology; Humana Press: Totowa, NJ, 2013; Vol. 1025, pp. 137–155 ISBN 978-1-62703-461-6.
